# Subjective evaluation of facial asymmetry with three-dimensional simulated images among the orthodontists and laypersons: a cross-sectional study

**DOI:** 10.1186/s12903-023-03167-9

**Published:** 2023-07-19

**Authors:** Mingjin Zhang, Liang Lyu, Jing Li, Huichun Yan, Yujia Zhu, Tingting Yu, Yong Wang, Yijiao Zhao, Yanheng Zhou, Dawei Liu

**Affiliations:** 1 Department of Orthodontics, Peking University School and Hospital of Stomatology, Beijing, China; 2 National Center of Stomatology & National Clinical Research Center for Oral Diseases & National Engineering Research Center of Oral Biomaterials and Digital Medical Devices, Beijing, China; 3 Beijing Key Laboratory of Digital Stomatology, Beijing, China; 4 Center of Digital Dentistry/Department of Prosthodontics, Peking University School and Hospital of Stomatology, Beijing, China; 5 NHC Research Center of Engineering and Technology for Computerized Dentistry Affiliation, Beijing, China

**Keywords:** Facial asymmetry, Three-dimensional images, Virtual face, Subjective evaluation, Visual analog scale (VAS)

## Abstract

**Objectives:**

We used three-dimensional (3D) virtual images to undertake a subjective evaluation of how different factors affect the perception of facial asymmetry among orthodontists and laypersons with the aim of providing a quantitative reference for clinics.

**Materials and methods:**

A 3D virtual symmetrical facial image was acquired using FaceGen Modeller software. The left chin, mandible, lip and cheek of the virtual face were simulated in the horizontal (interior/exterior), vertical (up/down), or sagittal (forward or backward) direction in 3, 5, and 7 mm respectively with Maya software to increase asymmetry for the further subjective evaluation. A pilot study was performed among ten volunteers and 30 subjects of each group were expected to be included based on 80% sensitivity in this study. The sample size was increased by 60% to exclude incomplete and unqualified questionnaires. Eventually, a total of 48 orthodontists and 40 laypersons evaluated these images with a 10-point visual analog scale (VAS). The images were presented in random order. Each image would stop for 30 s for observers with a two-second interval between images. Asymmetry ratings and recognition accuracy for asymmetric virtual faces were analyzed to explore how different factors affect the subjective evaluation of facial asymmetry. Multivariate linear regression and multivariate logistic regression models were used for statistical data analysis.

**Results:**

Orthodontists were found to be more critical of asymmetry than laypersons. Our results showed that observers progressively decreased ratings by 1.219 on the VAS scale and increased recognition rates by 2.301-fold as the degree of asymmetry increased by 2 mm; asymmetry in the sagittal direction was the least noticeable compared with the horizontal and vertical directions; and chin asymmetry turned out to be the most sensitive part among the four parts we simulated. Mandible asymmetry was easily confused with cheek asymmetry in the horizontal direction.

**Conclusions:**

The degree, types and parts of asymmetry can affect ratings for facial deformity as well as the accuracy rate of identifying the asymmetrical part. Although orthodontists have higher accuracy in diagnosing asymmetrical faces than laypersons, they fail to correctly distinguish some specific asymmetrical areas.

**Supplementary Information:**

The online version contains supplementary material available at 10.1186/s12903-023-03167-9.

## Background

Facial symmetry is commonly regarded as a key component of attractiveness [1]. However, studies have demonstrated that perfect facial symmetry does not exist in real people [2]. A small degree of bilateral facial asymmetry is observed in essentially all normal individuals [3–5], but this asymmetry is barely perceptible in daily life, and slight asymmetry may even create a more charming and harmonious appearance [6]. Patients with severe facial asymmetry, however, may suffer from both aesthetic and functional problems, which would exert negative effects on psychosocial development. Facial asymmetry also has a great impact on the patients’ diagnosis and treatment planning. With the advancement of treatment methods and increasing attention to the aesthetics of soft tissues, the diagnosis of facial asymmetry is becoming increasingly important. During the clinical examination, orthodontists can record soft tissue measurements and digitize a cephalometric radiograph or cone beam computed tomography (CBCT) to evaluate facial asymmetry [7, 8]. In recent years, some objective assessment tools, including three-dimensional face scans, have been developed to help clinicians define asymmetry [9]. However, most orthodontists prefer to examine patients’ faces with the naked eye to make quick judgements. Therefore, it is necessary to measure recognition accuracy to provide a better guide for clinical work.

Asymmetry occurring in different regions of the face has varying significance [3, 10]. When assessing soft tissue morphology, the lower third of the face has always been regarded as a key in orthodontic diagnosis and treatment. Therefore, symmetry of the chin, mandible and lip has naturally become a major clinical concern. The zygion region is regarded as a delicate component of the face, the protrusion of which is crucial to achieve smooth lateral facial aesthetic lines. Moreover, studies have shown that the cheek has higher asymmetry indices than other anatomical regions probably due to varieties of masticatory muscles [11]. Thus, in our study, we selected the chin (including the soft tissue of the mental tubercle and peripheral region), mandible (including the soft tissue gonion and peripheral region), lip (including the cheilion and peripheral region) and cheek (including the soft tissue zygion and peripheral region) to simulate in different directions and to study how different parts and directions might affect the perception of facial asymmetry.

Due to the increasing application and upgrading of 2-dimensional (2D) and 3-dimensional (3D) image processing software, many researchers began to deal with a variety of photos and collected the judgements through questionnaires or scoring to explore the identity of orthodontic aesthetic evaluation at the psychological level. It is critical to explore the subjective assessment of facial asymmetry in 3D system to provide a quantitative reference for clinics. Previous studies have mostly focused on defining a threshold of perception for facial asymmetry and perceived boundaries of facial deformity in different groups [2]. It was demonstrated that people could percept the asymmetry more easily and had more desire for surgery for greater asymmetries [2, 12, 13]. In addition, the professional groups including clinicians tended to be more rigorous to asymmetry than the laypersons [12, 14]. However, no studies have been done on the feature how people subjectively evaluate asymmetry in horizontal, vertical and sagittal directions as well as the potential confusion among different parts. Therefore, the aim of this investigation was to detect whether people can consistently identify the accurate asymmetrical part with both static and dynamic images and how degrees, types, and parts impact this process to provide quantitative references for clinics. In addition, the perceptions between orthodontists and laypersons were compared based on different aspects. The null hypotheses were that different observers assessed facial asymmetry similarly; and different degrees, types, and parts of asymmetry made no difference to subjective evaluation on facial asymmetry.

## Methods

### Study design

This is a cross-sectional study. The present study followed the Declaration of Helsinki on medical protocol and ethics, and the regional ethical review board of Peking University Hospital of Stomatology affiliated to Peking University, School of Medicine, approved the present study (approval number PKUSSIRB-202273044).

**Fig. 1:**
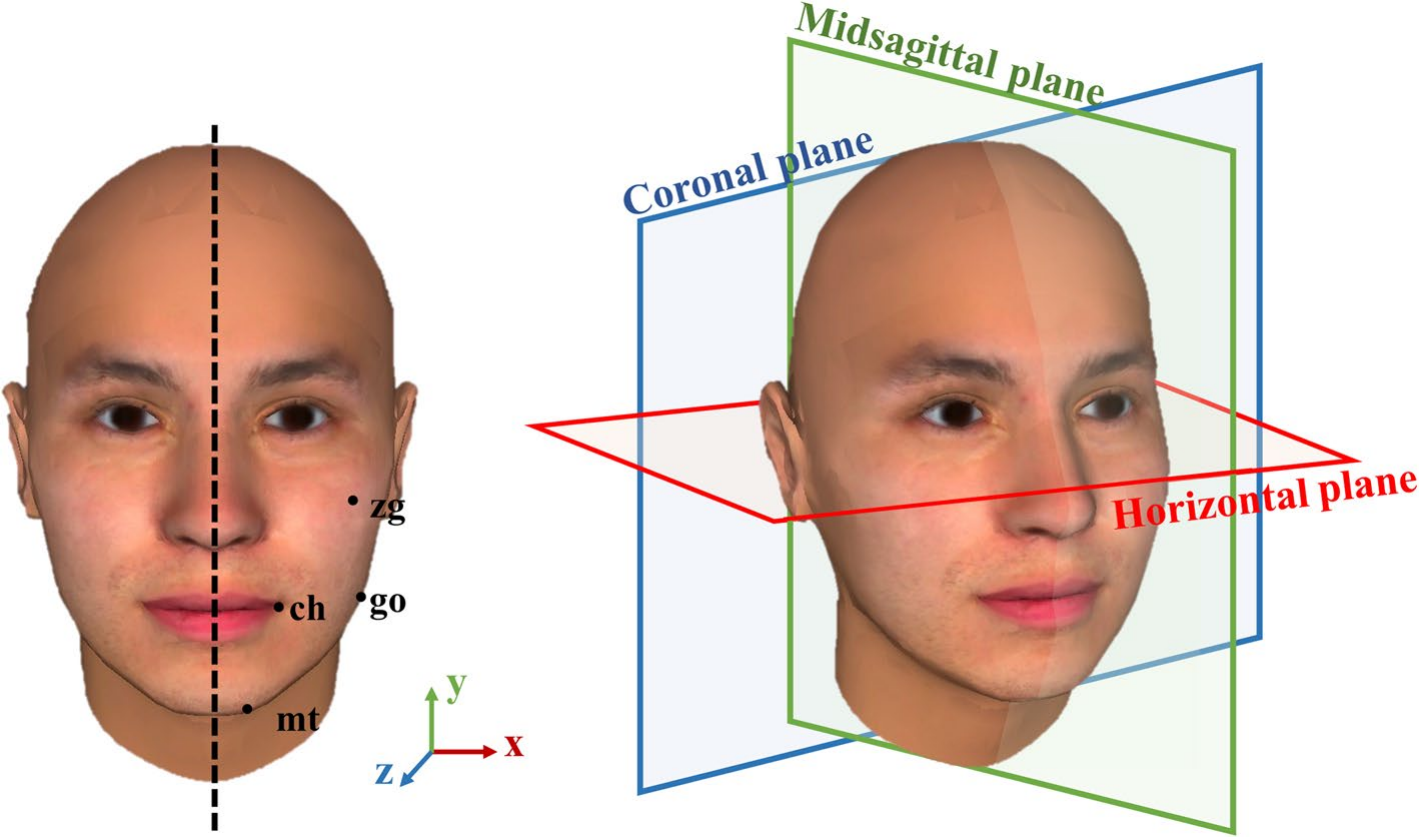
The symmetrical face template with display of facial anthropometric landmarks including cheilion (ch), soft tissue mental tubercle (mt), soft tissue gonion (go), and zygoma (zy) point and 3D reference planes (the horizontal plane, midsagittal plane, and coronal plane) shown on a lateral view of the symmetrical face

To illustrate irregular steric structures of the face, a stereoscopic “standard” face was constructed by FaceGen Modeller 3.4 (Singular Inversions Inc, Toronto, Canada) using proportion and soft tissue measurements based on parameters preset in the software. Bilateral facial parameters were adjusted to the same to create a “perfect symmetry” face as shown in Fig. 1.

**Fig. 2:**
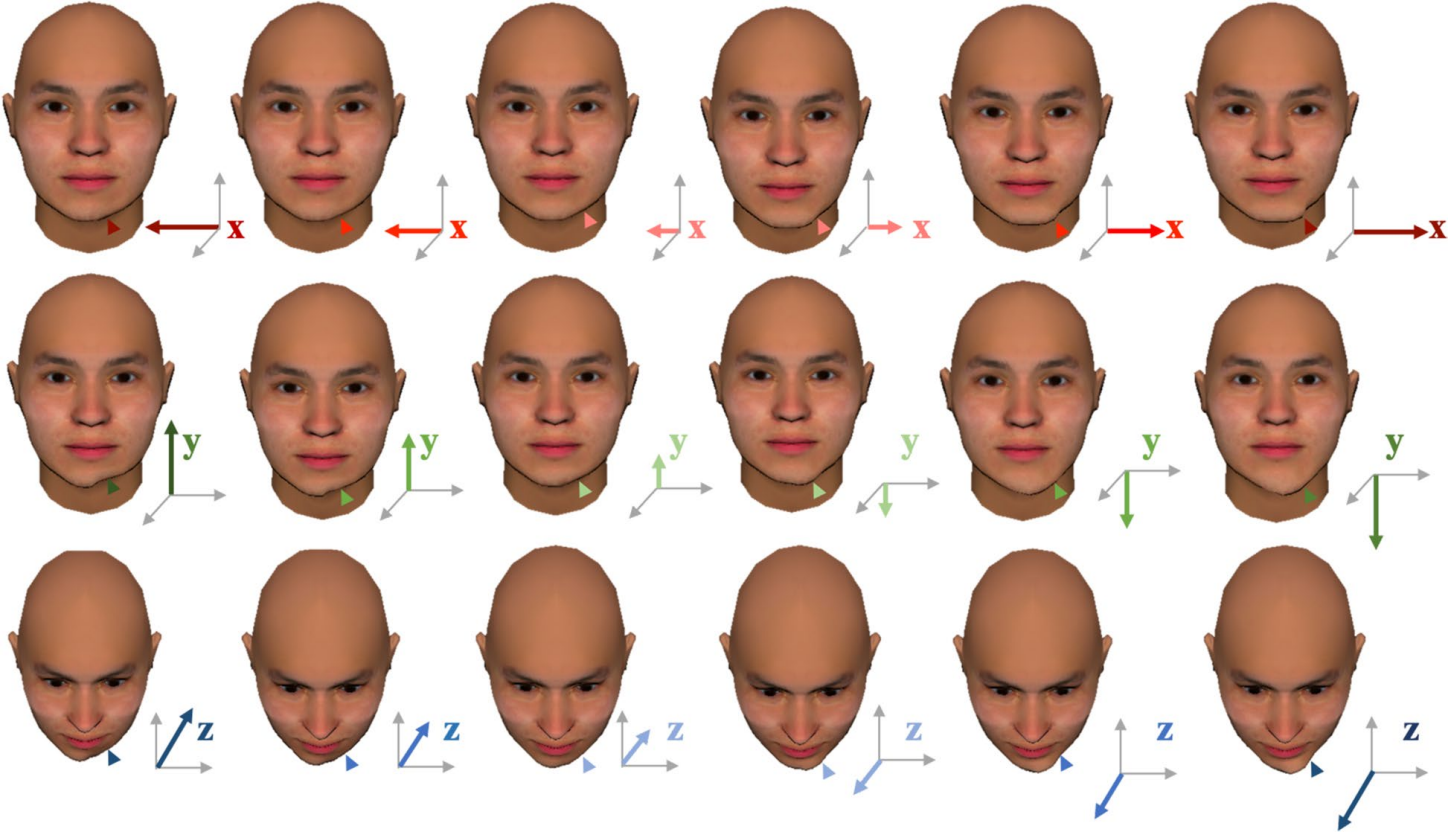
Chin asymmetry

A 3D coordinate system was established with the midsagittal plane perpendicular to the line that connected the bilateral medial canthus (Fig. 1). The horizontal plane was obtained by rotating the Camper’s plane (the plane consisting of bilateral tragion and midpoint of bilateral alare) upward 7.5 degrees [15]. The coronal plane was perpendicular to the median sagittal plane and horizontal plane. The standard face was processed to simulate deviation by Maya software (Autodesk Inc, San Francisco, CA, USA). For chin, mandible, lip and cheek asymmetry, the left cheilion (ch), soft tissue mental tubercle (mt), soft tissue gonion (go), and soft tissue zygion (zy) points and their peripheral regions were respectively manipulated to the left and right, up and down, and forward and backward respectively in 2-mm increments from 3 to 7 mm, in the horizontal, vertical and sagittal directions in the 3D coordinate system as we established in Fig. 1. Only the selected area of the left face was modified in every image, with the right face unchanged. An example of chin asymmetry is shown in Fig. 2. In the example, the change in the horizontal and vertical directions is displayed in the form of a frontal image and the change in the sagittal direction is displayed in a looking-down contour. The coordinate system bottom right suggests the direction in which the left mental tubercle was simulated. The color of the arrow represents the type of asymmetry (red represents the horizontal direction; green represents the vertical direction; blue represents the sagittal direction). The shade of color and the length of the arrow represent the degree of asymmetry (the darker the color is and the longer the arrow are, the more severe the asymmetry). The direction of the arrow represents the specific direction of asymmetry (interior/exterior, up/down, backward/forward).

### Setting and participants

Orthodontists and laypersons were selected as observers. A random recruitment of all raters was assured. The laypersons were recruited via an announcement online and orthodontists were selected at Peking University Hospital of Stomatology voluntarily. Orthodontists had to have had more than 2 years of clinical experience. The selection criteria for laypersons included the following: 1) age older than 18 years; 2) no orthodontic or plastic surgery-related experiences; 3) no serious facial deformity; and 4) no history of facial surgery.

### Measurement

**Fig. 3:**
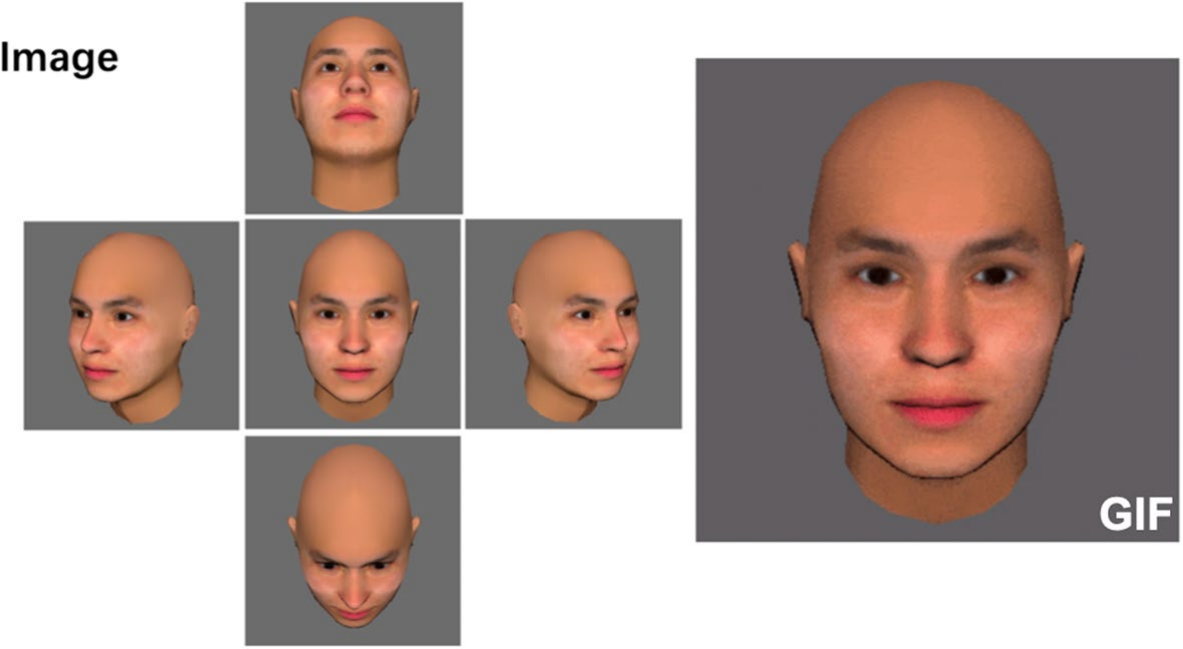
An example of an image viewed by study observers

Images (both.jpg and.gif files) in gray backgrounding were displayed in a fixed random sequence in a PowerPoint presentation (Microsoft, Redmond, WA) on the same computer. An example of the image viewed by the observers is presented in Fig. 3. Five multiangle pictures of 2D static images were produced to assist the overall perception of asymmetry, including the front face, left 45-degree profile, right 45-degree profile, looking-up contour at a 30-degree angle, and looking-down contour at a 30-degree angle. Each GIF was displayed for 15 s as an animation that started with a frontal view and then rotated from left to right and from up to down for visualization of the 3D feature of the face. Every page of PowerPoint would stop for 30 s for observers to evaluate the asymmetry and answer the questionnaire, with a two-second interval between pages. One increment was selected at random to be repeated twice to evaluate intraobserver agreement.

Each observer was invited to take part in the survey with a web-based survey service (https://www.mikecrm.com/). The participants were requested to provide the following information: sex (female/male) and age. A 10-point visual analog scale (VAS) was used to rate each image in asymmetry. An example of a section of the questionnaire is provided in Table 1.

**Table 1:** Section of questionnaire using 10-point VAS rating scale

**Image**	Rating (The more asymmetrical the face is, the lower the rank is.)		
	0 1 2 3 4 5 6 7 8 9 10		
**If you think the face is asymmetrical, please answer:**			
**Which part of the face do you consider not asymmetry?**	Mental tubercle (chin) Gonion (jaw) Cheilion (lip) Zygion (cheek) Others: ___	**Which direction do you think the part you chose has been changed?**	Left or right Up or down Forward or backward Others: ___

An instruction page accompanied the PowerPoint, with the following information: Some part of *the left face* has been changed/not changed from a completely symmetrical face to create an asymmetrical face, including mental tubercle (chin), gonion (mandible), cheilion (lip), zygion (cheek) and others. Only one part was changed per time in the horizontal (interior/exterior), vertical (up/down), or sagittal (forward or backward) direction. Please rate each image in terms of facial asymmetry from 0 to 10 in which 0 indicates the most severe asymmetry while 10 means no asymmetry. The more asymmetrical the face is, the lower the rank is. If you think the face is asymmetrical, please choose the part and direction you consider the transformation has been carried out (more than one part can be chosen).

### Study size

A pilot study was performed among ten volunteers to perform a power calculation. The sample size was calculated based on 80% sensitivity, with the anticipated standard deviations of rating at the 1.4 level. The mild asymmetry category was chosen as the base group and all other groups were compared to this. The minimum sample size to satisfy the test requirements was 30 observers per group. We increased the sample size by 60% of both orthodontists and laypersons in case the questionnaires were unqualified. All people from orthodontists group finished the questionnaire, and 8 people from layperson group were did not complete the survey and were excluded from the study. Therefore, a total of 48 orthodontists and 40 laypersons were included and analyzed in the present study ultimately.

### Statistical methods

SPSS software (Version 20, SPSS Inc) was used for data sorting and statistical analysis. Multivariate linear regression was used to assess the differences in symmetry ratings between the two groups (orthodontists and laypersons) with post hoc Bonferroni tests, adjusted for the concurrent effects of sex, age, degrees of asymmetry, type of asymmetry (horizontal, vertical or sagittal) and part of asymmetry (chin, mandible, lip or cheek). Multivariate logistic regression was used to assess the differences in the accuracy of identifying asymmetrical parts between the two groups and the independent variables included sex, age, work, degrees of asymmetry, type of asymmetry and part of asymmetry. The Spearman correlation coefficient was used to evaluate intraobserver agreement. A significance level of *p* < 0.05 was used.

## Results

The age and sex distributions of the observers are listed in Table 2. The Spearman rank correlation coefficient was 0.642 for the repeated increment, thus indicating moderate to good intraobserver agreement.

**Table 2:** Observer demographics

**Group**	**Sample** **Size (n)**	**Age**^a^ **(yr)**	**Female** **Gender (%)**
Orthodontists	48	27.19 ± 4.24	70.8
Laypersons	40	19.70 ± 1.47	47.5
Total	88	23.78 ± 4.97	60.2

^a^Data are presented as the mean ± standard deviation

### Ratings for asymmetry virtual face

In this study, we used ratings to measure how sensitive an individual was to face deformities. The ratings were inversely correlated with the severity of asymmetry people precepted subjectively. As observers’ rank dropped, the more severely they perceived asymmetry and thus the more sensitive they were to the type of asymmetry. Table 3 shows the descriptive statistics of asymmetry ratings of orthodontists and laypersons. Table 4 presents the results of multivariate linear regressions for the asymmetry rating, and Table 5 presents the results stratified by group.

**Table 3:** Descriptive statistics of asymmetry ratings of study groups

			**Orthodontists**									**Laypersons**								
**Part**	**Direction**	**Degree**	**Sample size**	**Mean**	**SD**	**95% CI**	**Min**	**First quartile**	**Median**	**Third quartile**	**Max**	**Sample size**	**Mean**	**SD**	**95% CI**	**Min**	**First quartile**	**Median**	**Third quartile**	**Max**
**mt**	**Horizontal**	**Mild**	96	6.73	2.04	6.30 to 7.16	2	5	7	9	10	80	6.99	1.61	6.63 to 7.33	3	6	7	8	10
		**Moderate**	96	5.34	1.78	5.00 to 5.71	1	4	5	7	9	80	6.24	1.81	5.84 to 6.65	2	5	6	8	10
		**Severe**	96	3.53	1.96	3.11 to 3.93	0	2	3	5	8	80	3.81	2.14	3.33 to 4.29	0	2	4	6	9
	**Vertical**	**Mild**	96	3.99	2.00	3.58 to 4.38	0	3	4	6	9	80	4.25	1.91	3.87 to 4.692	0	3	4	6	9
		**Moderate**	96	3.01	2.23	2.56 to 3.43	0	1	3	4	10	80	2.65	1.88	2.24 to 3.10	0	1	2	4	7
		**Severe**	96	1.56	1.73	1.24 to 1.90	0	0	1	3	7	80	1.35	1.44	1.04 to 1.67	0	0	1	2	5
	**Sagittal**	**Mild**	96	5.67	1.94	5.25 to 6.07	1	4	6	7	9	80	6.36	1.61	6.01 to 6.71	3	5	6	7	10
		**Moderate**	96	4.36	2.11	3.93 to 4.79	0	3	4	6	10	80	5.20	2.09	4.77 to 5.69	0	4	5.5	7	9
		**Severe**	96	3.46	2.15	3.05 to 3.90	0	2	3	5	9	80	3.83	2.22	3.35 to 4.29	0	2	4	5	8
**go**	**Horizontal**	**Mild**	96	5.85	2.29	5.40 to 6.35	1	4	6	8	10	80	6.56	1.83	6.15 to 6.92	2	5	7	8	10
		**Moderate**	96	4.20	2.31	3.73 to 4.66	0	2.25	4	5.75	10	80	4.44	2.19	3.93 to 4.93	0	3	4	6	10
		**Severe**	96	2.43	1.81	2.09 to 2.77	0	1	2	4	8	80	2.68	2.29	2.18 to 3.20	0	1	2	4	9
	**Vertical**	**Mild**	96	8.18	1.52	7.85 to 8.46	4	7	9	9	10	80	7.98	1.58	7.64 to 8.30	3	7	8	9	10
		**Moderate**	96	6.35	2.04	5.91 to 6.74	2	5	7	8	10	80	6.75	1.93	6.30 to 7.18	2	6	7	8	10
		**Severe**	96	5.13	2.06	4.72 to 5.56	1	3.25	5	6	10	80	5.69	1.79	5.30 to 6.06	1	4	6	7	10
	**Sagittal**	**Mild**	96	8.23	1.43	7.95 to 8.53	4	7	8	9	10	80	7.63	1.59	7.28 to 7.99	2	7	8	9	10
		**Moderate**	96	7.90	1.84	7.51 to 8.26	2	7	8	9	10	80	8.16	1.43	7.85 to 8.47	4	7	8	9	10
		**Severe**	96	7.11	2.03	6.71 to 7.50	2	6	7	9	10	80	7.30	1.73	6.92 to 7.69	0	6	8	9	10
**ch**	**Horizontal**	**Mild**	96	7.40	2.06	6.98 to 7.81	0	6	8	9	10	80	7.01	1.87	6.59 to 7.41	3	6	7	8.75	10
		**Moderate**	96	5.75	2.07	5.33 to 6.17	1	4.5	6	7.38	10	80	5.84	2.05	5.37 to 6.28	0	4.25	6	7.38	10
		**Severe**	96	4.10	2.24	3.60 to 4.58	0	2.25	4	6	10	80	3.60	2.30	3.08 to 4.12	0	2	3.5	5	10
	**Vertical**	**Mild**	96	6.92	2.16	6.48 to 7.36	0	5	7	9	10	80	6.13	2.10	5.64 to 6.61	1	5	6	8	10
		**Moderate**	96	3.71	2.58	3.18 to 4.24	0	2	3	5	9	80	3.45	2.53	2.92 to 4.06	0	1	3	5	10
		**Severe**	96	2.34	1.97	1.93 to 2.75	0	1	2	4	8	80	2.19	1.91	1.78 to 2.62	0	0.25	2	3	8
	**Sagittal**	**Mild**	96	7.78	1.58	7.46 to 8.08	3	7	8	9	10	80	8.10	1.49	7.76 to 8.42	5	7	8	9	10
		**Moderate**	96	7.20	2.00	6.81 to 7.60	2	6	8	9	10	80	6.81	1.84	6.43 to 7.24	2	6	7	8	10
		**Severe**	96	6.53	2.05	6.14 to 6.93	1	5	7	8	10	80	5.63	2.20	5.15 to 6.12	0	4	6	7	10
**zy**	**Horizontal**	**Mild**	96	7.36	1.86	7.00 to 7.75	2	6	8	9	10	80	7.54	1.47	7.22 to 7.89	4	6.25	8	9	10
		**Moderate**	96	5.85	2.44	5.31 to 6.36	0	4	6	8	10	80	6.09	2.03	5.64 to 6.54	1	5	6	8	10
		**Severe**	96	5.22	2.95	4.69 to 5.82	0	3	5	8	10	80	5.28	2.72	4.68 to 5.83	0	4	5	7	10
	**Vertical**	**Mild**	96	8.15	1.55	7.83 to 8.44	2	7	8.5	9	10	80	8.20	1.24	7.92 to 8.50	5	7	8	9	10
		**Moderate**	96	7.72	1.73	7.39 to 8.05	3	6	8	9	10	80	7.93	1.38	7.62 to 8.23	5	7	8	9	10
		**Severe**	96	7.48	2.11	7.05 to 7.88	1	6	8	9	10	80	7.43	1.53	7.09 to 7.74	3	6	8	9	10
	**Sagittal**	**Mild**	96	7.78	1.88	7.40 to 8.14	1	7	8	9	10	80	7.93	1.33	7.64 to 8.22	4	7	8	9	10
		**Moderate**	96	6.95	2.16	6.50 to 7.40	1	6	7	9	10	80	6.94	1.83	6.57 to 7.35	2	6	7	8	10
		**Severe**	96	6.16	2.45	5.66 to 6.66	0	5	7	8	10	80	6.33	1.95	5.86 to 6.73	0	5	7	8	10

**Table 4:** Multivariate linear regression analysis results for rating

Variable	Coefficient	95%CI	*p* value
Sex (female vs. male)	-0.358	-0.474 to -0.242	< 0.001
Age	0.058	0.041 to 0.075	< 0.001
Work (layperson vs. orthodontist)	0.431	0.260 to 0.601	< 0.001
Degree of asymmetry of image (mm)	-1.219	-1.286 to -1.151	< 0.001
**Level of degree of asymmetry of image**			
Moderate vs. mild	-1.250	-1.384 to -1.115	< 0.001
Severe vs. mild	-2.437	-2.572 to -2.831	< 0.001
**Type of asymmetry of image**			
Vertical vs. horizontal	-0.045	-0.179 to 0.090	0.514
Sagittal vs. horizontal	1.233	1.099 to 1.368	< 0.001
Sagittal vs. vertical	1.278	1.143 to 1.412	< 0.001
**Part of asymmetry of image**			
go vs. mt	1.907	1.752 to 2.063	< 0.001
ch vs. mt	1.261	1.106 to 1.416	< 0.001
zy vs. mt	2.676	2.520 to 2.831	< 0.001
ch vs. go	-0.646	-0.801 to -0.491	< 0.001
zy vs. go	0.768	0.613 to 0.924	< 0.001
zy vs. ch	1.414	1.259 to 1.570	< 0.001

*Abbreviation*: *CI* Confidence interval, *go* soft tissue gonion, *mt* soft tissue mental tubercle, *ch* Cheilion, zy soft tissue zygoion

**Table 5:** Multivariate linear regression analysis results for ratings stratified by group

Variable	Coefficient	95%CI	*p* value	Coefficient	95%CI	*p* value
	**Orthodontists**			**Laypersons**		
Sex (female vs. male)	-0.405	-0.572 to -0.237	< 0.001	-0.330	-0.492 to -0.168	< 0.001
Age	0.061	0.043 to 0.079	< 0.001	0.028	-0.027 to 0.083	0.326
Degree of asymmetry of image (mm)	-1.207	-1.301 to -1.114	< 0.001	-1.232	-1.329 to -1.136	< 0.001
**Level of degree of asymmetry of image**						
Moderate vs. mild	-1.307	-1.495 to -1.119	< 0.001	-1.181	-1.375 to -0.987	< 0.001
Severe vs. mild	-2.415	-2.603 to -2.227	< 0.001	-2.465	-2.659 to -2.271	< 0.001
**Type of asymmetry of image**						
Vertical vs. horizontal	0.063	-0.125 to 0.251	0.509	-0.174	-0.368 to -0.020	0.078
Sagittal vs. horizontal	1.28	1.091 to 1.468	< 0.001	1.178	0.984 to 1.372	< 0.001
Sagittal vs. vertical	1.216	1.028 to 1.404	< 0.001	1.352	1.158 to 1.546	< 0.001
**Part of asymmetry of image**						
go vs. mt	1.969	1.752 to 2.186	< 0.001	1.833	1.609 to 2.057	< 0.001
ch vs. mt	1.564	1.346 to 1.781	< 0.001	0.898	0.674 to 1.122	< 0.001
zy vs. mt	2.779	2.562 to 2.996	< 0.001	2.551	2.327 to 2.775	< 0.001
ch vs. go	-0.405	-0.622 to -0.188	< 0.001	-0.935	-1.159 to -0.711	< 0.001
zy vs. go	0.81	0.593 to 1.027	< 0.001	0.718	0.494 to 0.942	< 0.001
Zy vs. ch	1.215	0.998 to 1.432	< 0.001	1.653	1.429 to 1.877	< 0.001

*Abbreviation*: *CI* Confidence interval, *go* soft tissue gonion, *mt* soft tissue mental tubercle, *ch* cheilion, *zy* soft tissue zygoion

The results illustrated that the sex and age of the observer had a significant effect on the rating in total (Table 4). The female decreased the rating compared with the male, and the rating arose for each year increase in age of the observer. However, in multivariate linear regression stratified by group (Table 5), age lost significance among laypersons (*p* = 0.326).

The orthodontist group differed from the layperson group, giving a grading of 0.43 points lower in the VAS scale than laypersons, which meant the professional tended to be more critical of asymmetry (*p* < 0.001).

The degree of asymmetry had a great influence on the rating (*p* < 0.001). For each 2-mm increase in the asymmetry of the image, the observers decreased the rating, on average, by 1.219 on the VAS scale (Table 4).

The type of asymmetry of the image also had a significant effect on ratings (Table 4). Horizontal and vertical asymmetry decreased the rating compared with sagittal asymmetry (*p* < 0.001). However, no statistically significant difference was found between horizontal and vertical asymmetry (*p* = 0.514). The same results were shown in the multivariate linear regression stratified by group, which suggested that people were less sensitive to asymmetry in the sagittal direction (Table 5).

For the part of asymmetry of the image, chin asymmetry was given the lowest rating, followed by lip asymmetry, mandible asymmetry and cheek asymmetry. Specifically, chin asymmetry received on average 1.261 lower ratings than lip asymmetry, 1.907 lower ratings than mandible asymmetry, and 2.676 lower ratings than cheek asymmetry (*p* < 0.001). The differences among the ratings of these parts were all statistically significant from one another (*p* < 0.001) and were confirmed in both the orthodontist group and the layperson group (Table 5).

### Recognition accuracy of asymmetry virtual face

Except for symmetry ratings, we calculated recognition accuracy representing the likelihood of individuals successfully identifying the changed regions. The rating scale was dichotomized into 2 categories: if the observers managed to identify the asymmetry part, the accuracy point was marked as 1, while if they failed, the accuracy point was marked as 0. Multivariate logistic regression results for the binary outcome are presented in Table 6, and Table 7 displays the results stratified by group.

**Table 6:** Multivariate logistic regression analysis results for accuracy

Variable	OR	95%CI	*p* value
Sex (female vs. male)	1.085	0.965 to 1.221	0.172
Age	0.991	0.975 to 1.009	0.324
Work (layperson vs. orthodontist)	0.797	0.712 to 0.891	< 0.001
Degree of asymmetry of image (mm)	2.301	2.143 to 2.472	< 0.001
**Level of degree of asymmetry of image**			
Moderate vs. mild	2.657	2.321 to 3.041	< 0.001
Severe vs. mild	5.256	4.558 to 6.062	< 0.001
**Type of asymmetry of image**			
Vertical vs. horizontal	0.753	0.656 to 0.865	< 0.001
Sagittal vs. horizontal	0.429	0.374 to 0.493	< 0.001
Sagittal vs. vertical	0.570	0.498 to 0.653	< 0.001
**Part of asymmetry of image**			
go vs. mt	0.151	0.127 to 0.179	< 0.001
ch vs. mt	0.388	0.328 to 0.460	< 0.001
zy vs. mt	0.142	0.120 to 0.168	< 0.001
ch vs. go	2.574	2.208 to 2.999	< 0.001
zy vs. go	0.940	0.809 to 1.093	0.421
zy vs. ch	0.365	0.313 to 0.426	< 0.001

*Abbreviation*: *OR* Odds ratio, *CI* Confidence interval, *go* soft tissue gonion, *mt* soft tissue mental tubercle; ch, cheilion; zy, soft tissue zygoion

**Table 7:** Multivariate logistic regression analysis results for accuracy stratified by group

Variable	OR	95%CI	*p* value	OR	95%CI	*p* value
	**Orthodontists**			**Laypersons**		
Degree of asymmetry of image (mm)	2.308	2.093 to 2.546	< 0.001	2.315	2.084 to 2.571	< 0.001
**Level of degree of asymmetry of image**						
Moderate vs. mild	2.848	2.364 to 3.431	< 0.001	2.497	2.047 to 3.044	< 0.001
Severe vs. mild	5.277	4.339 to 6.418	< 0.001	5.343	4.332 to 6.590	< 0.001
**Type of asymmetry of image**						
Vertical vs. horizontal	0.748	0.619 to 0.905	0.003	0.756	0.618 to 0.925	0.007
Sagittal vs. horizontal	0.415	0.343 to 0.502	< 0.001	0.441	0.360 to 0.540	< 0.001
Sagittal vs vertical	0.555	0.460 to 0.669	< 0.001	0.583	0.477 to 0.712	< 0.001
**Part of asymmetry of image**						
go vs. mt	0.111	0.087 to 0.143	< 0.001	0.196	0.154 to 0.249	< 0.001
ch vs. mt	0.220	0.171 to 0.282	< 0.001	0.695	0.547 to 0.883	0.003
zy vs. mt	0.100	0.078 to 0.128	< 0.001	0.194	0.153 to 0.247	< 0.001
ch vs. go	1.976	1.609 to 2.425	< 0.001	3.553	2.820 to 4.477	< 0.001
zy vs. go	0.899	0.734 to 1.101	0.302	0.993	0.793 to 1.224	0.954
zy vs. ch	0.455	0.370 to 0.559	< 0.001	0.280	0.222 to 0.352	< 0.001

*Abbreviation*: *OR* Odds ratio, *CI* Confidence interval, *go* soft tissue gonion, *mt* soft tissue mental tubercle, *ch* cheilion, *zy* soft tissue zygoion

The sex and age of the observers showed no significant effect on the accuracy of the identification of asymmetric virtual faces (Table 6). Compared with the orthodontists’ group, the odds of the observers identifying the asymmetrical part correctly decreased by approximately 20% (*p* < 0.001), which indicated that orthodontists tended to identify the part of facial asymmetry more precisely than laypersons.

The degree and type of asymmetry were significantly associated with the accuracy of identification of facial asymmetry (Table 6). The odds were 2.301-fold greater for each 2-mm increase in the degree of asymmetry (*p* < 0.001). In addition, the odds were decreased by 24.7% for vertical asymmetry compared with horizontal asymmetry (odds ratio [OR], 0.753; *p* < 0.001) and 57.1% for sagittal asymmetry compared with horizontal asymmetry (OR, 0.429; *p* < 0.001). The overall trend illustrates that sagittal asymmetry is the most difficult to identify, while horizontal asymmetry is the easiest to recognize, and vertical asymmetry falls in between.

**Fig. 4:**
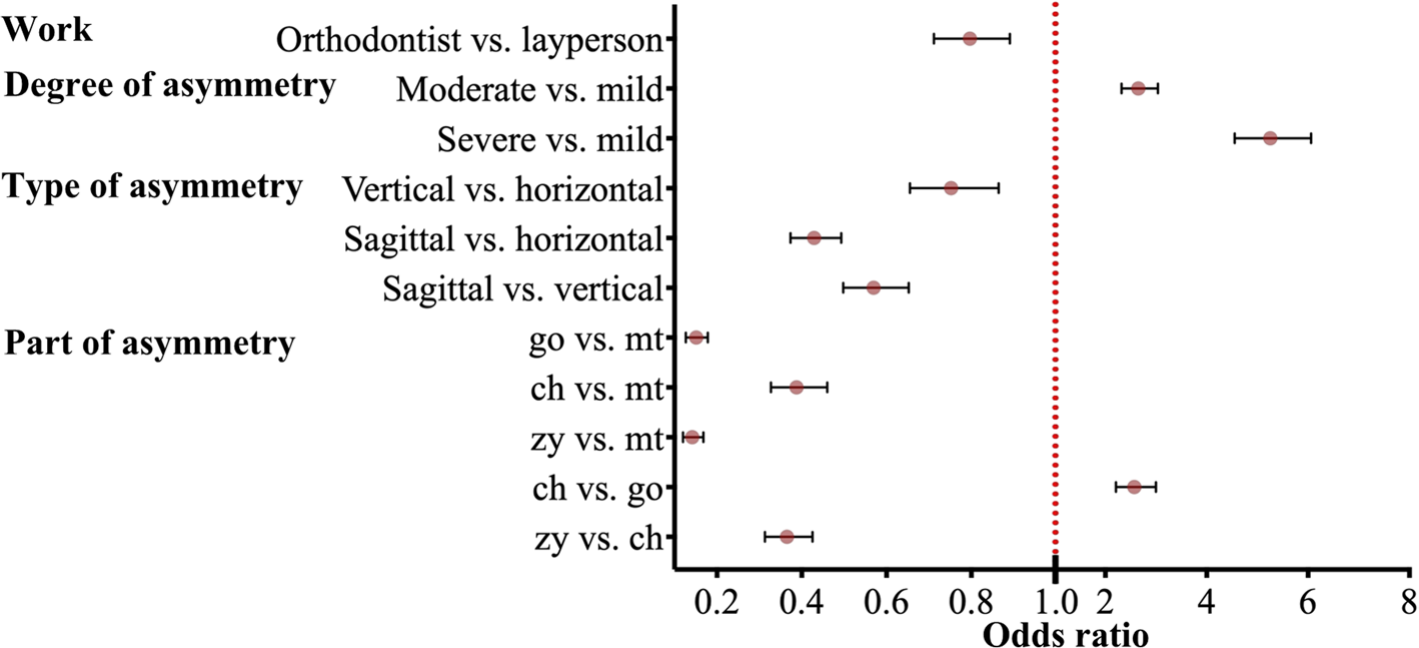
Odds ratio plot shown multivariate logistic regression analysis for accuracy

**Fig. 5:**
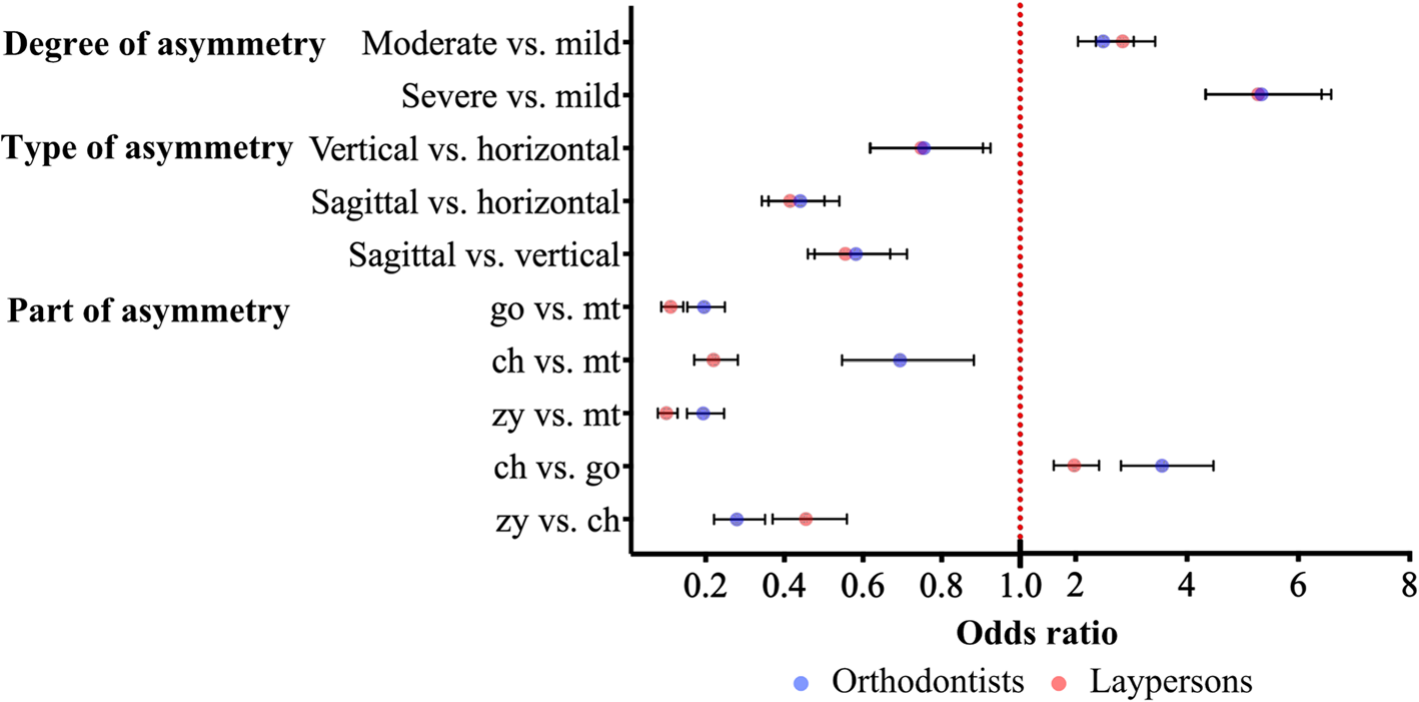
Odds ratio plot shown multivariate logistic regression analysis for accuracy stratified by group

For different parts of asymmetry, the odds were decreased by 84.9% for mandible asymmetry compared with chin asymmetry (OR, 0.151; *p* < 0.001), 61.2% for lip asymmetry compared with chin asymmetry (OR, 0.388; *p* < 0.001), 85.8% for cheek asymmetry compared with chin asymmetry (OR, 0.142; *p* < 0.001), and 63.5% for cheek asymmetry compared with lip asymmetry (OR, 0.365; *p* < 0.001). The odds increased by 2.574-fold for lip asymmetry than for mandible asymmetry (*p* < 0.001). However, the odds of perception between mandible asymmetry and cheek asymmetry showed no statistically significant difference (OR, 0.940; *p* = 0.421). The results showed a similar tendency in the accuracy of identification of the asymmetrical part when orthodontists and laypersons were considered separately (Table 7). The statistically significant results were demonstrated individually in odds ratio plots (Figs. 4 and 5). In summary, the recognition accuracy of chin asymmetry was the highest, followed by lip asymmetry, and the lowest were mandible and cheek asymmetry.

### Confusion of precepting different asymmetrical parts

To evaluate perceptual confusion when identifying different parts, the confusion matrices are shown in Table 8, with columns defining the true asymmetrical part and rows defining the part that the observers chose. The true asymmetrical part was classified into more specific types (horizontal [interior and exterior], vertical [up and down] and sagittal [backward and forward]) to thoroughly study the confusion of perception among different regions. The diagonal elements represent the recognition accuracy, and the off-diagonal entries correspond to the error rates.

**Table 8:**
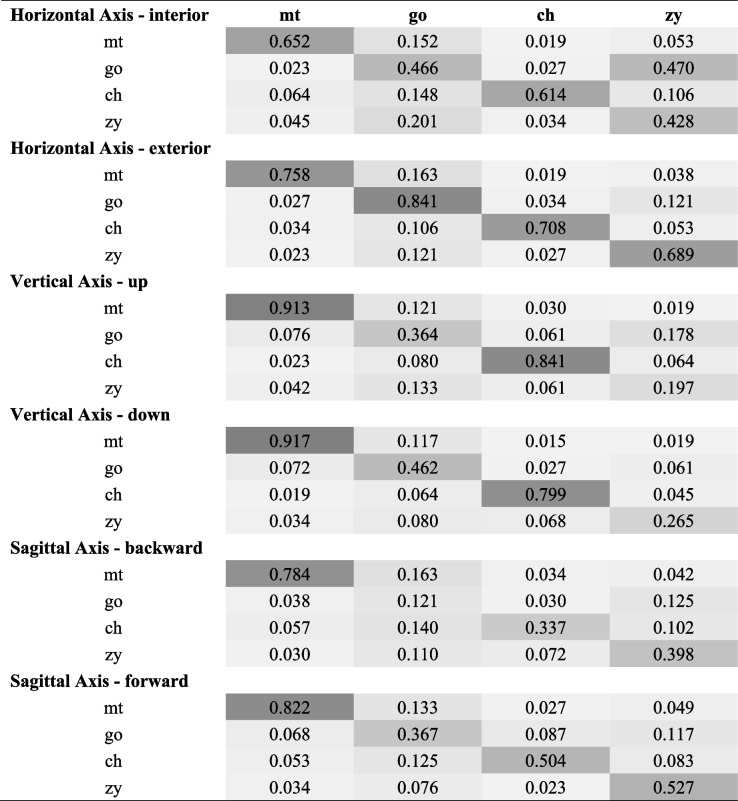
Confusion matrices

*Abbreviation*: *go* soft tissue gonion, *mt* soft tissue mental tubercle, *ch* cheilion, *zy* soft tissue zygoion. A grayscale color palette was used to color code the percentages of recognition/error rates. The darker the color was, the higher recognition/error rates were

The most obvious misjudgment is mandible asymmetry for cheek asymmetry in the horizontal direction when the gonion area is simulated to the interior (in our study, the left gonion area was simulated to the left horizontally). Even mandible asymmetry (recognition rate [RR], 0.466) was classified as cheek asymmetry (error rate [ER], 0.470) more often. In addition, when the gonion area was retruded asymmetrically in the sagittal direction (in our study, the left gonion area was simulated backward sagittally), mandible asymmetry (RR, 0.121) tended to be confused with cheek asymmetry (ER, 0.125) as well. Additionally, chin asymmetry maintained high recognition rates despite the types of asymmetry and was relatively confused for the mandible. Lip asymmetry could be readily distinguished in the horizontal and vertical directions but was confused in the sagittal direction by the mandible, cheek, and, to a lesser degree, chin. Cheek asymmetry had a low recognition rate in the vertical direction and was sometimes confused for the mandible, especially in the horizontal direction.

## Discussion

Facial asymmetry is mentioned as patients’ chief complaint; thus, it is vital that the region and type of asymmetry be precisely determined for accurate diagnosis, treatment planning, and communication with patients. To explore how different factors affect the subjective evaluation of facial asymmetry, we analyzed two aspects of information: the ratings and recognition accuracy for asymmetric virtual faces. Low ratings for the 3D face indicated that the asymmetry tended to be considered severe and easily perceived, while low recognition accuracy represented asymmetrical regions that were less noticeable and more difficult to distinguish in clinics.

In our research, it has been revealed that the age of the observer affected symmetry ratings which increased with growth in the age of the observer (Table 4). Dong et al. [12] also found that age had a significant effect on rating and older persons had the tendency to be more tolerant and conservative to facial asymmetry. Additionally, females seemed to be stricter to facial asymmetry, which might be explained by the fact that the females were slightly more sensitive to facial attractiveness. However, neither age nor sex showed a significant difference in recognition rate, which meant that these two factors had no influence on distinguishing asymmetrical parts.

Our study demonstrated that orthodontists not only were more sensitive to asymmetry but also had higher accuracy in diagnosing asymmetrical faces than laypersons. These conclusions were essentially in agreement with previous studies, some of which have also confirmed that orthodontists might be more rigorous to asymmetry and show a stronger desire for surgery under the same conditions [12, 16, 17]. The difference may contribute to the medical education and clinical experience of the professional group. Orthodontists are more likely to concentrate on recognizing and modifying facial asymmetry, especially in the lower third of the face, which is regarded as an important diagnosis project before the treatment and an evaluation indicator after the treatment.

For the degree of asymmetry, our results and those from other studies [12, 18] demonstrated that the greater the degree of asymmetry was, the more evidently and accurately observers could perceive the asymmetry (Tables 4 and 5). In our study, the recognition accuracy of different degrees of asymmetry indicated a rapid increase in recognition accuracy as the degree of asymmetry grew. Wang et al. [2] reviewed the previous studies evaluating the perception of progressive facial asymmetry in clinicians or laypersons using a 2D or 3D model and found that the threshold of precepting asymmetry was an abrupt, statistically significant increase in detection that could be best described by a sigmoid curve. Hohman et al. [19] determined that the identification of eyebrow elevation asymmetry gradually rose from 23% correct to 97% correct across the range of 1 mm to 6 mm of asymmetry. Asymmetry involving larger deformation could be expected to be identified more easily.

The type of asymmetry also has a considerable impact on people’s perception of facial asymmetry. Farbad et al. [20] concluded that horizontal asymmetry of the chin and mandible was less perceived than vertical asymmetry using 2D frontal facial images investigating orthodontists and laypersons. Our study demonstrated that horizontal and vertical asymmetry showed no statistically significant difference in asymmetry rating, while horizontal asymmetry was easier to distinguish than vertical asymmetry despite asymmetrical parts. The minor difference may result from the fact that we presented both 2D static images and 3D dynamic graphs, while the former study used 2D photographs, which could have affected the perception of asymmetry. In addition, for recognition accuracy, sagittal asymmetry is the least noticeable among the three directions possibly on account of people’s habits of precepting asymmetry in frontal images rather than profiles so that people are more likely to neglect sagittal changes.

Various parts of asymmetry seem to have distinct ratings and recognition accuracy. In our study, chin asymmetry obtained the lowest ratings, followed by lip asymmetry, mandible asymmetry and cheek asymmetry (Tables 4 and 5). On the other hand, chin and lip asymmetry are regarded as more discernible than mandible and cheek asymmetry, which means that under the same conditions, the former can be distinguished more accurately than the latter (Tables 6 and 7). Wu et al. [21] included three hundred and thirty 3D images of patients who were considered to have asymmetry and assessed by ten judges. Their results indicated that chin and lateral mandible deviation were significant factors affecting the diagnosis of facial asymmetry, which are also known to be most asymmetrical structures on the face [22]. Meyer-Marcotty et al. [23] assessed 3D perception of nose and chin deviation and found that alterations of nose were always judged as more asymmetric than identical aberrations of the chin, because it was suggested that nose was closer to the midline and was the longitudinal shale along the facial vertical axis. It has been reported that asymmetry has a larger influence near the midline, while in the marginal areas, minor asymmetric features might increase aesthetics [24]. This might explain why, in our study, people were more sensitive to chin and lip asymmetry in that these regions could be considered as closer to the midline, while the mandible and cheek are further.

We have studied the confusion tables as well, which indicate which parts are mistaken for others and how often in six different types of asymmetry (interior, exterior, up, down, backward and forward). We determined that chin asymmetry was easily recognized despite the type of asymmetry. Lim et al. assessed the self-recognition of facial asymmetry in skeletal Class III patients and found that menton deviation was a reliable diagnostic variable and a determinant in the recognition of facial asymmetry [25]. Lip asymmetry could be readily distinguished in the horizontal and vertical axes but was occasionally confused in the sagittal axis by the mandible. Mandible asymmetry was most often mistaken for cheek asymmetry especially when the mandible deviated to closer to the midline horizontally (Table 8). The protrusion of the zygomatic complex is on the turning point of the lateral contour, which forms facial aesthetic lines together with the soft tissue of the cheek and chin area. The zygion region (cheek) and gonial region (mandible) consist of the outer boundary of the face contour in frontal images. Therefore, the retraction of the gonial region may create an illusion that the zygion region has become more asymmetrical. However, the widened mandible (the gonial area simulated to exterior to midline) was rarely confused with the cheek, and cheek asymmetry was also less often mistaken for mandible asymmetry (Table 8). A possible explanation might be that the zygion area stands out more than the gonial area and distracts the attention to the mandible. The narrowed mandible might make the disparity more obvious, while the asymmetry of other conditions would be weakened or even concealed. In addition to the confusion discussed above, although backward movement of the mandible and cheek were similarly confusing (Table 8), the recognition rates were fairly low (approximately 12%), which made the conclusion less meaningful. Currently, few studies have discussed the interactions of perception between different regions. More cognitive features of how the naked eye recognizes facial asymmetry should be discovered in the future.

There are several limitations to this preliminary study. First, there should have been a higher level of heterogeneity of the sample in relation to the age group and gender of the participants, as most participants were aged less than 30 years in the present study. Besides, we used a Caucasian face as a template in this study. However, cross-culture difference of identifying own- and other-faces should also be considered in future research. Second, in addition to the factor we discussed about in this study, the orientation of facial asymmetry is also considered as an important factor in subjective evaluation. Haraguchi et al. found that 79.7% of subjects with facial asymmetry had a wider right hemiface and that 79.3% of the subjects with chin deviation showed left-sided laterality [26]. Meyer et al. found that a difference in the size of hemifaces could cause a bias toward the larger hemifaces [23]. Future studies should establish the role that left–right laterality plays in asymmetry perception to generalize the results. Third, only orthodontists and laypersons were chosen in our study, and there is disparity in aesthetics knowledge between these two groups. Testing a broader range of majors (i.e., plastic surgeons, general dentists) could extend the current findings in future studies. Also, the ratings in our study were not normally distributed using the Shapiro–Wilk and Kolmogorov-Smrinov tests. Further research should be carried out to obtain more universal conclusions.

### Future perspectives

In our present study, only one part was simulated in one direction per time. Actual clinical situations, however, could be much more complicated than they were in our study. There is a strong possibility that patients in reality have deviations in more than one part of the face in a variety of directions because the human face possesses a diversity of muscles on each side, which form a complex interdependent system to produce changes in the superficial geometry of the face and contribute to a wide range of functions [27]. For instance, mandible asymmetry may affect the depressor anguli oris, mentalis and depressor labii inferioris muscles, which are attached to the mandible and consist of the lip, and then stretch the orbicularis oris and the cutis to the deviated side, resulting in lip asymmetry [11]. Future research should examine more specific and complicated facial asymmetry to better imitate clinical conditions.

## Conclusions

The present analysis emphasizes that orthodontists seem to be more sensitive to asymmetry than laypersons. The degree, types and parts of asymmetry can all affect the subjective evaluation of facial deformity. Sagittal asymmetry is the least noticeable compared with horizontal and vertical asymmetry. Among these areas in our study, people were most sensitive to chin deviation, and mandible deviation is likely to be confused for cheek asymmetry especially in the horizontal axis. Although orthodontists have higher accuracy in diagnosing asymmetrical faces, they cannot always manage to distinguish the specific asymmetrical area correctly. More scientific and efficient examinations, rather than relying completely on subjective evaluation, should be developed to assist with clinical work.

## Supplementary Information

**Additional file 1: sFig.1.** Lip asymmetry. The coordinate system bottom right suggests how the left cheilion was simulated. The change in the horizontal and vertical directions is displayed in the form of a frontal image and the change in the sagittal direction is displayed in a looking-down contour. **sFig. 2.** Mandible asymmetry. The coordinate system bottom right suggests how the left gonion was simulated. The change in the horizontal and vertical directions is displayed in the form of frontal image and the change in the sagittal direction is displayed in a looking-down contour. **sFig. 3.** Cheek asymmetry. The coordinate system bottom right suggests how the left zygion was simulated. The change in the horizontal is displayed in the form of a frontal image and the change in the vertical and sagittal directions is displayed in a left 45-degree profile.

**Additional file 2.**

## Data Availability

The datasets generated and/or analyzed during the current study are not publicly available due to concerns that publication of information might impact participants’ provision but are available from the corresponding author on reasonable request.
