# Supplementary material for: Subjective evaluation of facial asymmetry with three-dimensional simulated images among the orthodontists and laypersons: a cross-sectional study
Source: BMC Oral Health. 2023 Jul 19;23:500. doi: 10.1186/s12903-023-03167-9 (PMC10354989; doi:10.1186/s12903-023-03167-9)
Supplement: Supplementary file 1 — Additional file 1: sFig.1. Lip asymmetry. The coordinate system bottom right suggests how the left cheilion was simulated. The change in the horizontal and vertical directions is displayed in the form of a frontal image and the change in the sagittal direction is displayed in a looking-down contour. sFig. 2. Mandible asymmetry. The coordinate system bottom right suggests how the left gonion was simulated. The change in the horizontal and vertical directions is displayed in the form of frontal image and the change in the sagittal direction is displayed in a looking-down contour. sFig. 3. Cheek asymmetry. The coordinate system bottom right suggests how the left zygion was simulated. The change in the horizontal is displayed in the form of a frontal image and the change in the vertical and sagittal directions is displayed in a left 45-degree profile. [file 12903_2023_3167_MOESM1_ESM.pdf]

Supplementary Material

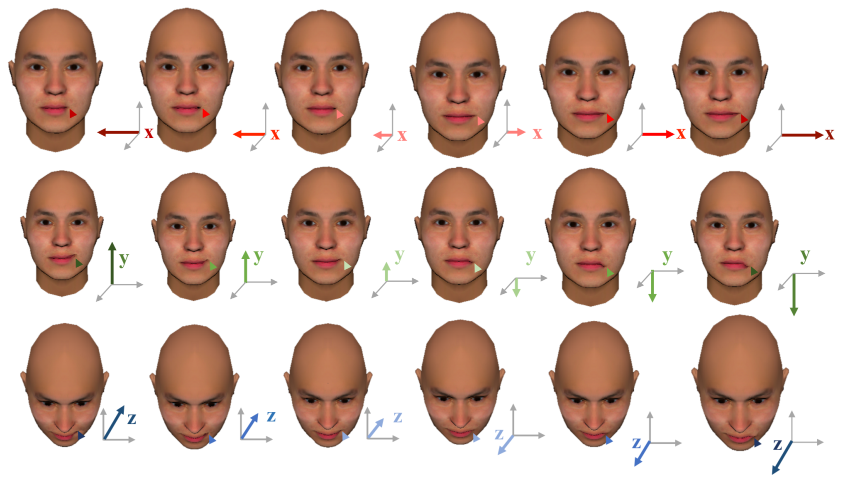

sFig.1. Lip asymmetry. The coordinate system bottom right suggests how the left cheilion was simulated. The change in the horizontal and vertical directions is displayed in the form of a frontal image and the change in the sagittal direction is displayed in a looking-down contour.

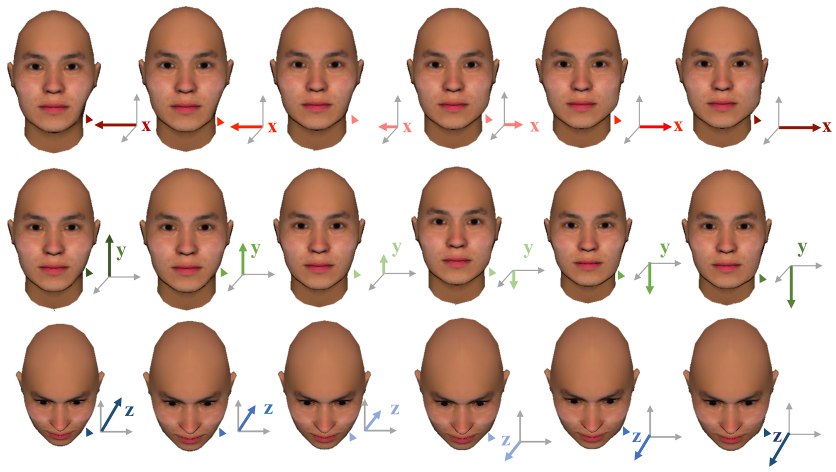

sFig.2. Mandible asymmetry. The coordinate system bottom right suggests how the left gonion was simulated. The change in the horizontal and vertical directions is displayed in the form of frontal image and the change in the sagittal direction is displayed in a looking-down contour.

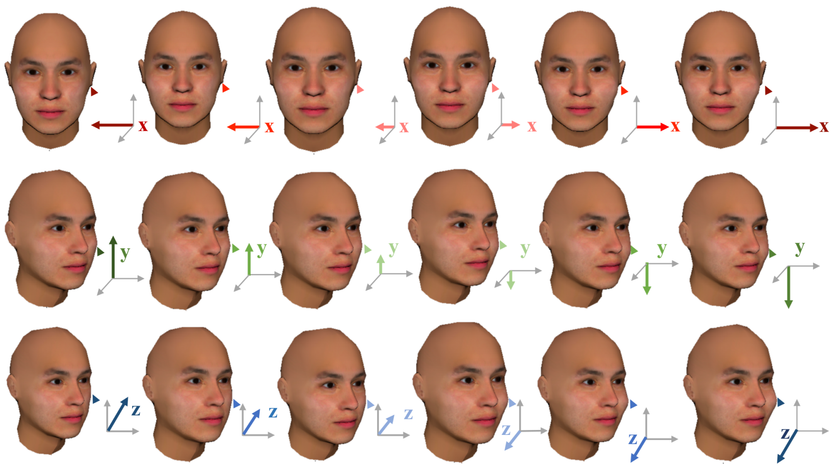

sFig.3. Cheek asymmetry. The coordinate system bottom right suggests how the left zygion was simulated. The change in the horizontal is displayed in the form of a frontal image and the change in the vertical and sagittal directions is displayed in a left 45-degree profile.
